# Notices

**Published:** 1863-01

**Authors:** 


### NOTICES.

**ROBERT CARTER & BROTHERS, No. 530 Broadway, New-York,**

Besides having the most extensive assortment of theological books, foreign and domestic, in this country, have issued for the holidays the following volumes, admirably adapted for the reading of the young; the publications of this old house are always safe, and calculated to promote the highest interests of society: "Tony Starr's Legacy, or Trust in a Covenant-Keeping God;" "Broad Shadows on Life's Pathway;" "Vesper," by the Countess De Gasparin; "Day-Break, or Right Struggling and Triumphant;" "The Bleak Cliff, and other Stories on the Parables;" "Mother's Last Words," a book of Ballads; "Bertie Lee;" "Ned Manton, or the Cottage by the Stream;" "Margaret Warner, or the Young Wife at the Farm;" "The Torn Bible;" "The Lost Jewel;" Little Walter of Wyalusing."

Of "Vesper," by the Countess de Gasparin, of "Margaret Warner," and the "Torn Bible," published, as above noted, by Robert Carter & Brothers, New-York, a cotemporary well says: "'Vesper' is rich in fancy, a poem in prose raiment, simple, touching, with a vein of delicate humor. It is full of the charm of domestic life, and flushed with the gentle warmth of a pure and womanly religion. It will be read with a healthy delight by young and old. 'Margaret Warner' is a delightful little story of home life, which leaves the heart full of loving-kindness and of tranquil joy. 'The Torn Bible,' a story of a soldier's life, and well adapted to lead the soldier, and the young who shall peruse it, to that precious spring of all good, the Word of our God." There was sold in London, in a short time, no less than seventy thousand copies of Mrs. Sewell's "Mother's Last Words," being ballads for boys and girls.

THE following was received December 5th, 1862, purporting to come from "Andrew Miller, attorney and counselor-at-law, No. 206 South Fifth street, Philadelphia." The writer, or any other subscriber, who feels aggrieved in the same way, will have their dollar returned by returning to our office the twelve JOURNALS received for 1862:

"W. W. HALL, M.D., NEW-YORK CITY:

"SIR: I have received the number for this month of your JOURNAL OF HEALTH, which completes the volume for 1862, for which I have paid, and I beg you will discontinue sending the paper to my address. When I subscribed for it, I supposed it to be what its title indicated, namely, a JOURNAL OF HEALTH; but I find it to be a journal for the dissemination of the most vile and disgusting abolitionism. The September number I threw in the fire after reading a few paragraphs of the leading article headed 'A Sick Nation;' and I think any one who would send to those who had paid for a journal of health such treasonable abolitionism as is contained in that article, ought to be prosecuted for obtaining money under false pretenses."

That our sentiments may not be mistaken as to the great question of the times, we put upon record the following, to be brought up in the future for good or ill as to ourself and our children: As I consider that slavery is the cause of this war, I earnestly hope it will never close until means have been devised in righteousness, by Congress, for freeing, soon and forever, every slave on this continent, and for a complete and eternal crushing of the great rebellion, in the shortest time possible, by all the means which God and nature and right and law have, or may yet, put into the hands of the true friends of the Government of the United States; and then our beloved country, in the enjoyment of a free press, free speech, free religion, all under the molding influences of the Bible, and an intelligent democracy, is destined to a career of material prosperity, of national power, and of moral grandeur, unknown and undreamed of in all the ages past.

W. W. HALL,
Editor of HALL'S JOURNAL OF HEALTH.

HYGIENIC HOUSE, 170 Bleecker street, New-York.—Persons of sedentary employment who desire to live where a greater variety than usual of bread, fruit, and grain preparations are offered, and less of highly-seasoned food, will find this the place. A greater variety than usual of bread, fruit, and grain preparations, and less of meats and highly-seasoned food, will form the distinctive feature of our table. Terms—$5 to $12 per week, according to accommodations required. Transient board $1 per day. Meals may be had at the regular hours. Breakfast or Tea, 25 cents each. Dinner, 35 cents. Hours of Meals—Breakfast 7 to 8. Dinner 1 to 2½. Supper 6 to 7.

W. HUNT,
R. FANCHER.

N. B.—The Bleecker-street stages pass the door. The location is within three or four minutes' walk of the Sixth Avenue cars, or of Broadway, and ten minutes' walk of Cooper Institute, Astor, or Mercantile Libraries.

SKATING IN FIFTH AVENUE AND FIFTY-SEVENTH STREET.—A Lake of eleven acres. Oscar F. Oatman, Esq., Superintendent. Season Tickets, $5. Gentleman and two ladies, $10. John L. Brown, General Superintendent. Under the two gentlemen named, the public have a guarantee that every thing connected with this famous resort of fashion and fun will be handsomely managed.

HARPER'S WEEKLY, Pictorial, $2; Harper's Monthly, $3; Both, $4 a year.

### NOTICES.

ATLANTIC MONTHLY, published by Ticknor & Fields, No. 135 Washington street, Boston, Mass.; three dollars a year; Volume 11 began with January. Among its contributors are some of the best intellects and the most cultivated minds in the nation. It is the great literary monthly of the country, and by the acknowledged ability with which it has been conducted, it has been placed on a permanent basis, and is highly appreciated abroad as well as at home.

THE HORTICULTURIST, founded by the lamented A. J. Downing, in 1846, is in its eighteenth volume: two dollars a year; No. 37 Park Row, New-York. Its patronage is commended to country gentlemen and intelligent agriculturists throughout the country.

THE PRESBYTERIAN, Philadelphia, two dollars and fifty cents a year, has an industrious and able correspondent in this city; his weekly letters well pay for the subscription-price to every New-Yorker who wishes to keep himself posted as to the "goings on" and chief doings of our mighty metropolis.

TO FARMERS.—There is no monthly published on this or any other continent on agriculture or on any other subject which gives one half as much valuable, practical, and reliable information for one dollar a year as the *American Agriculturist*, issued at No. 7 Park Row, New-York City.

BLACKWOOD'S MAGAZINE, two dollars a year, *London Quarterly*, *The Edinburgh*, *The Westminster*, and *North British Reviews*, each three dollars, are all furnished for ten dollars a year. Address Leonard Scott & Co., No. 54 Gold street, New-York. The contributors to these publications are among the very ablest writers in Great Britain.

MUSIC.—The hinged-plate piano improvement of Horatio Wooster, of New-York, is eliciting the admiration and hearty commendation of the most accomplished artists in the country. Among the names are those of Gottschalk, Muzio, Mason, Berge, Fredel, Thomas, Harrison, Wernike, Morgan, Gosché, and the distinguished amateur Dr. Thomas Ward, all substantiating the sentiment of Gottschalk, when he said, "I estimate the volume of tone to be increased one hundred per cent by this invention," which is certainly very high praise, coming as it does from the very highest musical authority.

GODEY'S LADY'S BOOK, three dollars a year, Philadelphia, continues as heretofore to be the Queen of pictorial monthlies, delighting multitudes of families with its beautiful steel engravings and its valuable practical embellishments, etc.

ARTHUR'S HOME MAGAZINE, two dollars each year, Philadelphia. Who that has ever subscribed for it, ever willingly failed to "renew" when Christmas came?

PILES, FISTULA, RUPTURES, ETC.—The last published work of Dr. Bodenhemer on these and kindred subjects has been translated into French. Dr. B. spends the winter at the Monongahela House, Pittsburgh, and for knowledge, ability, skill, and success has no superior living.

TEETH.—Dr. John Allen, 22 Bond street, New-York, in whose office Dr. Evans, now the first Dentist in Europe, took lessons is believed to be the ablest member of his profession for furnishing single and sets of teeth. We know cases where twenty years of youthfulness have been imparted to the features.

THE Farm-House Milk, pure and sweet, is brought to town daily by the New-Jersey and Rockland County Milk Association, under the management of C. W. Canfield, Esq., No. 146 Tenth street, New-York, near Broadway, adjoining Stewart's New Retail Palace.

BARNUM'S MUSEUM continues to be the general place of resort for novelty-seekers. Formerly a "Museum" was considered to be a collection of all the queer, outlandish things of creation, but Mr. Barnum, with characteristic energy and forecast, has made his establishment a place not only of amusement but of solid instruction. Scarcely a week passes in which some new object of interest is not introduced. Natural treasures are gathered from the poles to the tropics; yesterday he had a polar bear; to-day a family of Esquimaux; to-morrow it will be a whale, or a multitude of fishes of all sizes and hues, from the Pole to the Line; and frequently all are seen at once, exciting the mind of the beholder alternately with feelings of awe, of wonder, of admiration and delight.

IRON FENCES, railings, plain and ornamental, statues, figures of animals, bedsteads, gate-posts, tree-guards, with every conceivable variety of article for families, farms, cemeteries, parks and pleasure-gardens and grounds, are found at the very extensive establishment of Hutchinson & Wickersham, 259 Canal street, New-York, one of the oldest and best known houses of the kind in the city.

WE heartily commend "The Home Monthly," two dollars a year, Boston, to every household wishing a whole year of delightful and instructive reading for wives, husbands, daughters, and sons.

